# Sustainable mitigation of heavy metals from effluents: Toxicity and fate with recent technological advancements

**DOI:** 10.1080/21655979.2021.1978616

**Published:** 2021-09-27

**Authors:** Vivek Kumar Gaur, Poonam Sharma, Prachi Gaur, Sunita Varjani, Huu Hao Ngo, Wenshan Guo, Preeti Chaturvedi, Reeta Rani Singhania

**Affiliations:** a Amity Institute of Biotechnology, Amity University Uttar Pradesh, Lucknow Campus, Lucknow, India; b Department of Bioengineering, Integral University, Lucknow, India; c Department of Microbiology, Indian Institute of Management and Technology, Aligarh, India; d Paryavaran Bhavan, Gujarat Pollution Control Board, Gandhinagar, Gujarat India; e Centre for Technology in Water and Wastewater, School of Civil and Environmental, Engineering, University of Technology Sydney, Sydney, NSW – Australia; f Aquatic Toxicology Laboratory, Environmental Toxicology Group, Council of Scientific and Industrial Research-Indian Institute of Toxicology Research (Csir-iitr), Lucknow Uttar Pradesh, India; g Department of Marine Environmental Engineering, National Kaohsiung University of Science and Technology, Kaohsiung City, Taiwan

**Keywords:** Heavy metals, biochar, sensors, bioremediation, sustainability

## Abstract

Increase in anthropogenic activities due to rapid industrialization had caused an elevation in heavy metal contamination of aquatic and terrestrial ecosystems. These pollutants have detrimental effects on human and environmental health. The majority of these pollutants are carcinogenic, neurotoxic, and are very poisonous even at very low concentrations. Contamination caused by heavy metals has become a global concern for which the traditional treatment approaches lack in providing a cost-effective and eco-friendly solution. Therefore, the use of microorganisms and plants to reduce the free available heavy metal present in the environment has become the most acceptable method by researchers. Also, in microbial- and phyto-remediation the redox reaction shifts the valence which makes these metals less toxic. In addition to this, the use of biochar as a remediation tool has provided a sustainable solution that needs further investigations toward its implementation on a larger scale. Enzymes secreted by microbes and whole microbial cell are considered an eco-efficient biocatalyst for mitigation of heavy metals from contaminated sites. To the best of our knowledge there is very less literature available covering remediation of heavy metals aspect along with the sensors used for detection of heavy metals. Systematic management should be implemented to overcome the technical and practical limitations in the use of these bioremediation techniques. The knowledge gaps have been identified in terms of its limitation and possible future directions have been discussed.

## 1. Introduction

Heavy metals are metallic elements of the periodic table with characteristic high density. They occur naturally in the earth’s crust and yet are poisonous at low concentrations [1,2]. The pollution caused by heavy metals has become a global concern disturbing the environment and causing serious human health hazards [3,4]. Rapid urbanization and industrialization have been the root cause behind increasing heavy metal pollution. The increasing population, economic globalization, and industrial revolution have exponentially increased the diversity of contaminants [5]. Anthropogenic activities have drastically affected the geochemical cycle of heavy metals. Some of these metals viz., manganese (Mn), iron (Fe), cobalt (Co), copper (Cu), and zinc (Zn) are essential for human body in low concentrations however metals such as mercury and lead has no known beneficial effect [3,6]. Among the heavy metals arsenic (As), mercury (Hg), chromium (Cr), lead (Pb), and cadmium (Cd) are of most concern as these are non-threshold toxins which are reported to be present in higher concentration in the aquatic, terrestrial and aerial system [6,7]. Recently it was estimated that Hg, Pb, Cr, and Cd from different sources has posed a serious threat to 66 million people globally [6]. Furthermore, the water contamination by As has alone affected >150 million people globally [8]. The use of chemicals for metal removal further adds up to the environmental burden with the existing life-threatening situation posed by heavy metals, and the physicochemical process also does not provide a complete solution to the problem. Therefore, biological techniques employing the use of plants and microorganisms are being preferred owing to their environmental friendly and economical approaches [6,9,10].

Reduction or removal of toxic heavy metals has become a challenging task. There are four different methods to treat heavy metals namely *in-situ* treatment, ex-situ treatment, in-situ containment, and ex-situ containment. Based on these methods, the process of removal of heavy metals can be classified into chemical, physicochemical, and biological methods [11]. Various bacterial and fungal species such as *Pseudomonas aeruginosa, Paenibacillus jamilae, Bacillus subtilis, Aspergillus* sp, *Botrytis* sp, *Neurospora* sp, *Saprolegnia* sp, *Penicillium* sp, and *Trichoderma* sp. has been reported to actively metabolize and reduce different heavy metals [12–14]. Plants remove heavy metals by different processes such as *Typha latifolia, Brassica juncea*, and *Chara canescens (phytovolatilization)* [15,16] *Morus alba* and *Populus alba (phytoaccumulation/phytoextraction)* [9] *Helianthus annuus and Phaseolus vulgaris (rhizofilteration)* [9,17]. Recently bioaccumulation of several metals including cadmium (0.011), lead (0.047), arsenic (0.23), copper (0.92) and mercury (0.36) in mg/kg wet weight was reported in European eels muscle tissues [18]. It was found that the concentration of mercury was above the threshold limit prescribed by Water Framework Directive Environmental Quality Standards. Not only in the organisms but the heavy metals such as arsenic, chromium, mercury and cadmium was also found in the fine fraction of Kudjape landfill biocover. These metals may exhibit mobility and leaching potential yet the concentration of metals were below the values set by Estonia regulation [19].

For the detection of heavy metals the utilization of atomic absorption spectroscopy [20], inductively coupled plasma-mass spectroscopy (ICP-MS) [21], and atomic fluorescence spectrometry (AFS) [22] had limitations and thus poses difficulty for onsite detection. Therefore, the development of sensors to detect the traces of heavy metals in soil, drinking water, biological fluid, and food offers a sensitive, reproducible, and accurate procedure for environmental surveillance [23]. A portable biosensor for detection of bivalent mercury and trivalent arsenic traces in water was developed by Sciuto et al., 2021. The technology utilized engineered E.coli which selectively produced 4‐aminophenol upon selective metal interaction. The sensor was reported to exhibit a detection potential of 1.5 ppb (LOD), 5 ppb (LOQ), and 0.122 μA/ppb (sensitivity) for arsenic and 0.1 ppb (LOD), 0.34 ppb (LOQ) and 2.11 μA/ppb (sensitivity) (Sciuto et al., 2021).

Furthermore, very recently advancement has been done pertaining to the removal of heavy metals and these advancements include the use of biochar, biosurfactant, and biocatalytic removal [24–26]. Biosurfactants are secondary metabolites produced by microorganisms [27,28] which has been thoroughly reported for its potential to solubilize diverse xenobiotics pollutants including heavy metals [29,30]. In previous years, microorganism-based and plant-based remediation work has been excellently reviewed [31]. However, there is a scarcity of literature compiling the recent interventions on heavy metal remediation. Thus, here we have detailed the phyto- and micro-based heavy metal remediation approaches along with the recent advancements in reduction and sensors for detection of heavy metals. The knowledge gaps have been identified in terms of its limitation and possible future directions have been discussed.

## 2. Sources and environmental implications of heavy metals

Heavy metals have been reported with characteristics such as high density and severe toxicity. They are well-known environmental pollutants that include cadmium (Cd), arsenic (As), chromium (Cr), lead (Pb), and mercury (Hg). These are naturally occurring in the earth’s crust and enter the environment by different natural and anthropogenic sources and their exposure causes adverse effects on humans and animals [32–34]. Several natural sources were reported to spread heavy metals which include weathering of minerals, sea-salt sprays, volcanic activity, erosion, forest fires, aerosol particulates, and biogenic sources [35]. Anthropogenic phenomena which can spread arsenic contamination are agriculture, industrial wastewater, leather tanning, mining, metallurgical processes, burning of fossil fuels [33,36,37].

The use of pesticides, insecticides, and fertilizers are the main reasons behind the pollution caused by heavy metals through agricultural processes. Other major sources include automobile exhaust which contains Pb, smelting process releasing As, zinc (Zn), copper (Cu) [38]. These metals exist in the form of organic and inorganic compounds like hydroxides, silicates, and oxides. The source of toxic heavy metals is provided in Table 1. Arsenic is one of the well-known heavy metals that can be detected at low concentrations in the environment. Inorganic forms of arsenic are tetravalent and pentavalent whereas organic forms are monomethylarsonic acid, trimethylarsine oxide, and dimethylarsinic acid [39]. Arsenic has been used for manufacturing rat poison, herbicides, and pesticides. Gallium arsenide was used for detection purposes in x-rays and transistor technology. Organic arsenic was found in some foods like fish and shellfish and was used in cosmetics [37,40].

**Table 1.** Sources, toxicity and permissible limit of heavy metals by USEPA

Heavy metals	Anthropogenic Sources	Toxic effect	Permissible limit (drinking water)
Arsenic (As)	Metal processing, mining sites, timber storage, coke ovens emission, pesticide and fertilizer industries.	Accumulates inside the cell, Carcinogen	0.05 mg/L
Chromium (Cr)	Fertilizers, fossil fuels burning, oil drilling sites, metal tanneries and plating industries.	Group 1 human carcinogen	0.05 mg/L
Cadmium (Cd)	Phosphate fertilizers, battery, and plating industries.	Nephrotoxicity and Carcinogenicity, affect gastrointestinal and pulmonary tract	0.005 mg/L
Mercury (Hg)	Battery industries, pig iron, caustic soda, gold, and cement production	Chromosome breakage, bronchitis asthma and Hunter–Russell syndrome	0.002 mg/L
Nickel (Ni)	Electroplating industries	Eczematous reaction	0.1 mg/L
Copper (Cu)	Electroplating industries	Kidney damage, anemia	1.3 mg/L
Selenium (Se)	Mining, agricultural irrigation	Bronchitis, gastrointestinal disturbances	0.05 mg/L
Lead (Pb)	Mining, batteries, use of lead products	Crosses the blood–brain barrier, carcinogenic, neurodegeneration	0.05 mg/L
Iron (Fe)	Metal refining, engine parts.	Seizures or coma.	0.3 mg/L
[^32,33,42,43]^			

Heavy metals have become a global concern owing to their abundance and production during industrial processing and use to meet our needs. Different environmental compartments viz., soil, water, and air are severely affected by heavy metals [37]. The toxic heavy metals present in drinking water are lead, iron, copper, cadmium, zinc, and chromium. These metals are also required by the body, but in large amounts they become poisonous. Metals and their ionic forms possess chemical toxicity like irreversible mutation, damage to the main central nervous system, oxidative stress, muscular and neurological degeneration, and carcinogenicity in the liver and kidney when exposed for long term [41]. The concentration below which then tends to be safe is the permissible limit. The permissible limit for different heavy metals as given by the US-Environmental protection agency (USEPA) is given in Table 1. Exposure to heavy metals like cadmium, arsenic, mercury, and lead adversely affects the health of humans. Long-term exposure to these metals can result in neurological, muscular, and physical damage [33,42]. These metals were well known to affect cellular organelles such as nuclei, endoplasmic reticulum, cell membrane, and some enzymes that are involved in metabolism. The U.S national toxicology program along with the international agency for research on cancer concluded the evidence that cadmium is a human carcinogen [37]. Cadmium exposure in humans was reported to occur through several ways such as inhalation, cigarette smoking, and engulfment of foods that contain cadmium in trace amounts. Cadmium is fatal for humans, irritates the gastrointestinal and pulmonary tract (Table 1) which causes symptoms like vomiting, salivation, muscle cramps, etc. [43]. Exposure to chromium was reported to cause multi organ toxicity such as cancer of the respiratory tract, allergy, and asthma. Chromium (º) induced histopathological, genotoxic, biochemical effects in the kidney and liver of goldfish [32,37].

Furthermore, acute lead exposure may lead to brain damage, kidney damage, and it can also adversely affect vitamin D metabolism, kidney, and blood pressure. Lead was identified as a potential carcinogen in animals and cause tumors in rats and mice [37]. Another toxic heavy metal, Mercury imposes toxicity in form of elemental mercury vapor (Hgº), inorganic mercury, mercuric (Hgº[2]), and mercurous (Hgº[1]). All forms of mercury were toxic and spread adverse effects in humans which include gastrointestinal toxicity, nephrotoxicity, and neurotoxicity [33,37].

Heavy metals were reported to affect the physiological parameters of plants by causing biochemical and ultrastructural changes [44]. Cadmium affects plants by inhibiting their growth, nutrient uptake, photosynthesis, and root injury [45]. In cauliflower, cobalt stress affects nutrient uptake, transpiration rate, enzyme activity, chlorophyll content [46]. This suggested that heavy metal contamination has become a global concern with the knowledge of its increasing toxic effects and thus it has become imperative to address this problem through research advances.

## 3. Fate of heavy metals at organism level

**Figure 1.**
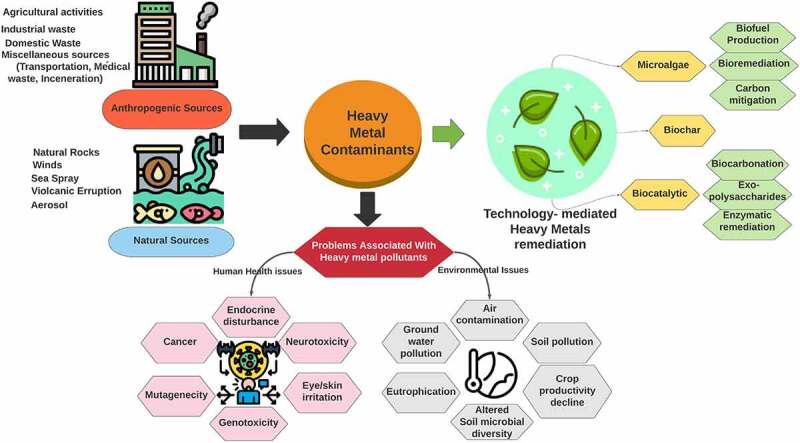
Schematic depicting the sources effects and remediation strategies for heavy metals

Heavy metals even at low concentrations were reported to cause severe health hazards owing to their gradual accumulation [47,48]. Schematic for sources effects and remediation strategies for heavy metals is shown in Figure 1. As discussed in the previous section that heavy metals enter the environment through several routes, thus it is of paramount importance that these can be completely removed or their toxicity is reduced. To reduce the toxicity, chelators were used in physical or chemical remediation. Alternatively, in microbial- and phyto-remediation the redox reaction shifts the valence making them less toxic [49]. This section detailed the microbial- and phyto-remediation strategy as an economic and environmentally friendly approach against heavy metal contamination.

*3.1 Microorganisms*: Metal cations are needed by microbial cells for various metabolic activities, however, high concentration was reported to form internal cell complexes and thus inhibit the growth *[46,50]*. Microorganism has the potential to remediate toxic heavy metals and, in this process, they did not generate toxic by-products *[51]*. Microorganisms respond differently to different heavy metals under different conditions. Bacteria can mobilize, transform, uptake, and immobilize heavy metals upon interaction *[46]*. Sequestration, exclusion, detoxification, and complexation were the major mechanisms followed by microbes *[52]*. Siderophores produced by the bacterial cells form complex with heavy metals and thus removes their toxic effects by limiting their bioavailability *[46,53]*. It was reported that the bacterial metabolites and transporters exhibit the potential of heavy metal detoxification. The metal ions thus compartmentalized inside the bacterial cells are detoxified by the sequestration method *[50,53]*.

Desoky et al., [14] isolated heavy metal tolerant bacteria from contaminated soil and studied them for their ability to reduce the toxicity of Cd and Pd on a spinach plant. Three bacterial species namely *Pseudomonas aeruginosa* DSM 1117, *Paenibacillus jamilae* DSM 13815 T, *Bacillus subtilis* subsp. spizizenii DSM 15029 was found to be effective. The concentration of Pb and Cd was recorded to be 38.1, 23.8 mg/kg and 17.1, 13.6 mg/kg in roots and leaf of metal stressed plant whereas the addition of *Bacillus subtilis* reduced the concentrations of Pb to 7.36 and 1.89 mg/kg in roots and leaves respectively, whereas Cd was 4.33 mg/kg in roots and traces were seen in leaves. Similarly, the supplementation with *P. jamilae* and *P. aeruginosa* was found effective in reducing the toxicity of Cd and Pb. The addition of these bacterial strains under the heavy metal stressed conditions helped in restoring the net photosynthesis, rates of transpiration, membrane stability index, relative water content, and stomatal conductance in spinach plant [14].

Additionally, fungal species were reported to physicochemical interact and sequester heavy metals to the cell surface [54]. The high content of cell wall material i.e. the presence of diverse metal binding functional groups elevates the fungal efficiency to sequester metal. Several fungal species such as *Aspergillus* sp, *Botrytis* sp, *Neurospora* sp, *Penicillium* sp, *Saprolegnia* sp, and *Trichoderma* sp, were employed for the removal of toxic heavy metals [12,13]. Four different fungi isolated from a scrap dumpsite by enrichment were found to effectively remove different heavy metals when studied in synthetic media. All the four isolates were reported to remove 10–20% of Cd (100 mg/L) and Hg (50 mg/L), 34–62.74% of As (10 mg/L) whereas more than 99% removal was recorded for Pb (50 mg/L) [12]. Pb and Cd from contaminates soils were effectively removed by *Saccharomyces cerevisiae*. It was recorded that 65–79% of heavy metals were biosorbed in 30 d [55]. Furthermore, the treatment of *Pinus massoniana* tree with ectomycorrhizal fungi significantly contributed to the survival of the plant while reducing the translocation of heavy metals [56]. It was recorded that the root of plants containing ectomycorrhizal fungi such as *Suillus* had high concentration of heavy metals whereas the shoots contain significantly low as compared to the plants with no ectomycorrhizal fungi in rhizosphere, suggesting its role in reducing the phytotoxicity [56,57]. Alternatively, it was observed that different factors *viz*., type and abundance of ectomycorrhizal fungi, heavy metal type, and plant adaptation to fungi exhibited different effects on the transport and absorption of heavy metals [57–59].

*3.2 Plants*: The plant-based technology used for the reduction or removal of pollutants is termed Phytoremediation *[60]*. In this technology either genetically modified or raw plants were used for restoring contaminated sites *[32]*. It offers a low-cost solution to the remediation process. Heavy metals upon interaction with plants can undergo different processes such as phytovolatilization, phytodegradation, phytoextraction, and phytoaccumulation, rhizofiltration, phytostabilization, phytodesalination, and rhizodegradation *[9,32,61]*.

**Figure 2.**
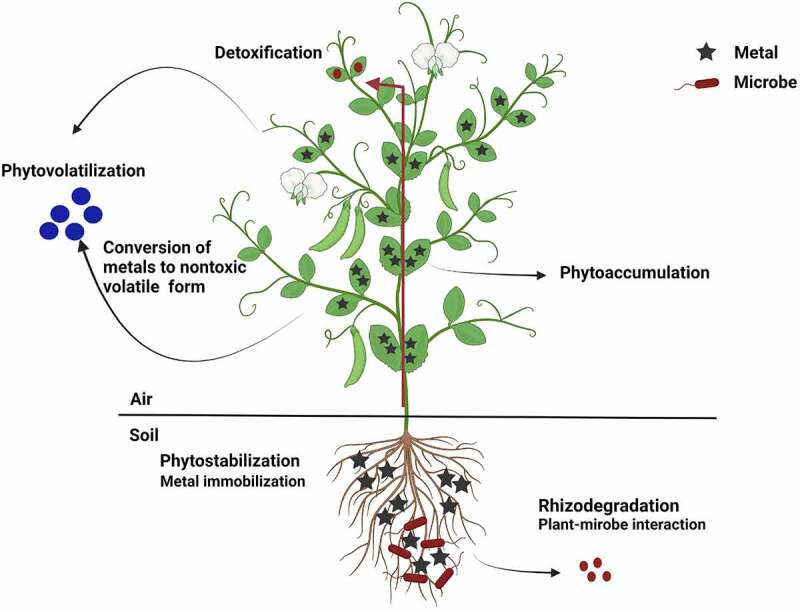
Figure depicting metal accumulation in plants

In the phytovolatilization process, plants uptake heavy metal contaminants and released to the atmosphere in a less toxic form (Figure 2) such as selenium converted to dimethyl selenide and mercury to mercuric oxide which was further volatilized [15]. Most of the plants were reported to volatilize dimethyl selenide whereas the presence of boron and sulfate (present as co-contaminate) inhibits the process. During phytovolatilization of selenium by plants, they were cultivated with crop rotation generating biomass for their use as a livestock feed supplement [32]. An aquatic plant *Typha latifolia* L. exhibited phytovolatilization and was effectively utilized for the remediation of selenium contaminated soil. *Brassica juncea* and *Chara canescens* were reported to absorb mercury and selenium followed by its release by volatilization [16]. Phytovolatilization offers a permanent solution for these contaminants owing to the fact that volatilized products are less likely to deposit at the same site.

During the phytoaccumulation or phytoextraction process, the plants uptake the contaminants from water or soil and accumulate them in shoots and leaves. Hyperaccumulator species having high potential to accumulate contaminants and produce biomass are preferentially utilized in this process [9,62]. Also, plant species exhibiting less accumulation but high biomass production can be utilized for the phytoextraction process. These plants were incinerated after phytoextraction and the ash can be disposed of by landfilling this led to the removal of contaminants from soil [9]. Rafati et al. [63] investigated the potential of *Morus alba* and *Populus alba* to uptake different heavy metals from soil. It was recorded that fallen leaves showed maximum accumulation of Ni and Cr in *M. alba* whereas Cr and Cd were high in *P. alba* in comparison to green leaves. In both the species the Cr metal at a treatment of 240 and 480 mg/kg was found to get transported to leaves and stem. However, in the phytostabilization process the mobility of heavy metals were arrested by their adsorption and precipitation in roots and rhizosphere respectively. The plant species used in this process amend the soil chemistry and thus facilitate the precipitation and adsorption of heavy metals [64]. Interestingly, during phytostabilization plants secrete redox enzymes that converts heavy metals to a less toxic form in soil [9]. This process only stabilizes and inactivates the heavy metals and thus can be employed as a management strategy [65]. The accumulation of heavy metal was studied in *Populus tremula* and *Picea abies* [66]. The long-term exposed plants showed up to 20 times more accumulation of heavy metals in fine roots as compared to the control. It was shown that the fine roots of *Picea abies* accumulated more metals than *Populus tremula*. The total accumulated heavy metal was 0.2% of the total amount of heavy metal in the soil [66].

Similar to this, rhizofiltration process works by adsorbing the contaminants to the root. This process can effectively reduce the level of heavy metals from surface water, groundwater, and contaminated wastewater. For this process, terrestrial plants were preferred owing to their developed root and fibrous system along with other characteristics such as metal tolerance, and hypoxia tolerance [17]. *Helianthus annuus and Phaseolus vulgaris* removed uranium from contaminated groundwater with greater than 90% efficiency. A major drawback to this was that the plants utilized had to be disposed of after they attain maximum adsorption of contaminants [9,17].

Phytodegradation is the process wherein the plants break down the contaminants into lower metabolites or less toxic forms, which can be utilized for plant growth. The breakdown may involve metabolic processes or enzymes [32]. In addition to the degradation that occurs in the rhizosphere with the aid of microorganisms was termed rhizodegradation [9,67]. The process of microbial aided degradation of heavy metals is detailed in the previous section.

## 4. Recent advances in sustainable removal of heavy metals

Considering the ill effects of heavy metals, it is an imperative need of time to explore a rapid and efficacious method to abolish these hazardous pollutants from soil and water bodies [68]. Biological methods are environmentally friendly, low-cost means to answer the persistent challenge of heavy metal toxicity in the environment.

*4.1 Biocatalytics*: Biocatalysts are biological weapons efficient in the chemical transformation of organic and inorganic components. Enzymes secreted by microbes and whole microbial cell are considered an eco-efficient biocatalyst for mitigation of heavy metals from contaminated sites. The microbial enzyme was reported to perform biocarbonation of heavy metals. The urease enzyme secreted by microorganisms decomposes urea into ammonium ions and carbonates. Then, carbonates form an insoluble complex with heavy metals in the process of biocarbonation and were found efficient in the reduction of heavy metals from contaminated soil *[69]*. The heavy metal carbonate complexes formed around microbes give rise to a condition of stress to the microbial cell. Heavy metal removal activity of bacterial urease was dependent upon strain. Plant derive urease enzyme (PDUE) was also useful in biocarbonation process, it promotes heavy metal precipitation and urea hydrolysis as microbial ureases without creating stress like conditions for soil microbiota *[70]*.

Plant exopolysaccharides (EPS) are tangled arrangements of high molecular weight microbial homopolysaccharides and heteropolysaccharides along with some other carbohydrates, protein, and metallic ions like Fe, K, Mg, and Mn as major constituents. Microbial EPS were reported to show a biosorption mechanism for detoxification of heavy metal contaminated laden soil. EPS are negatively charged therefore they attract positively charged heavy metal ions to forms a complex. EPS had emerged as an excellent scavenger of heavy metals with several lucrative benefits including low cost, sustainable and eco-friendly nature [71].

*4.2 Microalgae*: Microalgae were found to thrive in heavy metal loaded environments and efficiently participate in the removal of heavy metal contaminants via the biosorption process. The cell wall of microalgae possesses a unique complex structure, metal-binding proteins, and functional groups (carboxyl or amino groups) which provides a site of attachment to heavy metal ions. Biosorption is an inexpensive, simple, and eco-friendly process as compared to the conventional treatment process and does not produce any toxic by-products or toxic gases [72–74]. Besides biosorption microalgae are highly efficient in the bioaccumulation and biodegradation process to accomplish the detoxification process. Microalgae had extensive combination of extracellular and intracellular mechanism due to which they can tolerate the toxicity imposed by heavy metals, extend wide support, and suitability in bioremediation of contaminated site. Microalgae were found to be an eco-friendly multifunctional organism that can simultaneously be utilized for multiple technologies like carbon mitigation, bioremediation, and biofuel production [75].

Microalgae are rapidly growing microbes capable of producing biofuel by utilizing nutrients from wastewater [76]. They play a dual role in phytoremediation and bioenergy synthesis with a huge emphasis on green energy production [77]. *Chlorella vulgaris* was reported to be used as biosorbent in powder form to ease transportation and its application [78]. Lu et al. studied the detoxification potential of *Chlorella vulgaris* and reported that at 0.5 and 1.0 mg/L Cr(VI) concentration, the growth of *C. vulgaris* was significantly increased due to the antioxidant capacity of intercellular superoxide dismutase and catalase enzyme. While at higher Cr(VI) concentrations of 2.0 mg/L and 5.0 mg/L, the level of malondialdehyde, degree of cellular oxidative damage increases which further retards *C. vulgaris* growth in the batch culture process. During the continuous process in a membrane photobioreactor 50% of Cr reduction was obtained in 3 d for hydraulic retention time and 40 d for solid retention time at a maximum volumetric Cr removal rate of 0.21 mg/L/d [79].

Microalgae based bio-adsorbents developed by the thermochemical transfiguration of *Chlorella sorokiniana* biomass into graphitic bio-chars showed proficient results in heavy metal detoxification. Graphitic bio-chars had shown a synergistic impact on biomass production and wastewater decontamination in aquatic ponds [80]. Abhinandan et al., [81] cultivated two acid-tolerant microalgae namely *Heterochlorella* sp. MAS3, and *Desmodesmus* sp. MAS1 at pH 3.5 for mitigation of heavy metals such as Cu, Fe, Mn, and Zn and simultaneous biodiesel production. It was recorded that 10–20 mg L^−1^ concentration of Cu supported the growth of microbes and 40–60% of heavy metal removal was achieved. In situ transesterification of biomass in the vicinity of heavy metal ions yield enhanced biodiesel recovery. These findings suggested that microalgae were promising and suitable candidates for curing metal-rich acid mine drainages and sustainable production of biodiesel [81]. Incorporation of a hybrid system containing *C. vulgaris* coupled with calcined eggshells in acid mine drainage for heavy metal removal culminated conclusive outputs. In a panel photobioreactor, the biomass production was marked ~8.04 times higher than its initial concentration of 0.367 g/L, and light transmittance of 95% at 305 mm was achieved in 6 d incubation along with a significant 99.47 to 100% reduction in the heavy metal contaminants namely Cu, Fe, Cd, Mn, As, and Zn from the effluent [82].

## 5. Biosensors for heavy metal detection

Heavy metals are the contaminant of environmental and biological concern, owing to their non-biodegradable, prolonged half-life, and toxic nature [83]. This concern has triggered the development of sensors to detect the traces of heavy metals in soil, drinking water, biological fluid, and food to better understand the physiological and pathophysiological effects of heavy metals [84]. Conventional methods like atomic absorption spectroscopy [20], inductively coupled plasma-mass spectroscopy (ICP-MS) [21], microprobes [85], and atomic fluorescence spectrometry (AFS) [22] had some limitations such as high operating cost, expensive cost of the instrument, complex pre-processing of samples, and long detection time, which make them applicable in laboratory condition and difficult for onsite detection. Therefore, the development and implementation of sensors based technology in terms of sensitivity, reproducibility, portability, the limit of detection, and accuracy are highly desirable for environmental surveillance [23]. However, many different sensors with unique advantages are designed till date using synthetic biology approaches. Different platforms of sensors offer a great opportunity as they provide rapid, sensitive, and accurate detection of heavy metals in a meaningful way which makes the interpretation much easy. Development of optical, electrochemical, fluorescent, and nanoparticle sensors with multiplexed detection ability of heavy metals pushed the research area from design to application [86,87]. In response to entailing the onsite detection of lead (Pb) in blood and urine samples, a field deployable micro-analyzer based on flow-injection/stripping voltammetry (ASV) was developed which prevents electrode fouling. The device manifested finest sensitivity for Pb detection, which exhibited linear concentration of Pb exposure in humans i.e. up to 20 ppb in 10% of blood samples and 50ppb in 50% of urine. Unlike the ICP-MS, this miniature device requires very low concentration of reagent (Hg), thus minimizing the health concern associated with it [88]. Similarly, Boron doped diamond (BDD) electrode, a carbon-based material coupled with microelectrodialyser was used for the detection of Pb. The device allowed the detection of Pb^2+^ ions within a linear range from 20 ppb to 100 ppb with LOD of 19 nM in aqueous solution [89]. The same device was further employed to detect the Cd, Pb, Cu, and Zn [90]. Immediate detection of active traces of As in food and water chain is imperative as it exhibits destructive effects in humans and animals. Therefore, an extensive electrogenerated nanotextured gold assemblage (Au/GNE) was developed which allows the detection of ultra low level of As^3+^ up to 0.08 ppb in water. This sensor showed high sensitivity for As detection with a LOD of 0.1 ppb (1.3 nM) (derived from calibration curve) and 0.08 ppb (derived from linear regression). Au/GNE was incredibly applicable for the detection of As in the system containing Fe^2+^, Hg^2+^, Pb^2+^, Ni^2+^, Cu^2+^, and other metal ions [91]. Cadmium exposure to humans was reported to exhibit neurological and cardiovascular degeneration [92]. Selective detection of this contaminant is very crucial. In this regard, the carbon paste electrode was modified by using lanthanum tungstate ion exchanger to develop a sensor for the detection of Cd(II) ions. This sensor exhibited a Nerstian response under a working range of 8 × 10^−^[8] to 1 × 10^−1^ mol/L. It worked with a short response time of approximately 5 sec thus can be flexibly employed for 22 weeks without leaving any error, thus holds potential determination to estimate Cd (II) in solutions [93]. Another sensor employed to detect the mercury and its traces using colorimetric detection integrated with the antibody–antigen on the testing strip [94]. This portable strip was very effective having linear concentration in a range of 1 and 10 ng/mL, with LOD of 0.23 ng/mL. The accuracy of this immunological assay based sensor was evaluated by exogenously adding Hg(II) in water at different concentrations. The recovered sample was found in the range from 103.2% to 108.7%, thus it was useful for rapid monitoring at the site [94]. Additionally, Neupane and coworkers synthesized a device based on fluorescent peptidyl chemosensor to detect the traces of mercury. The peptidyl chemosensor bears tetraphenylethylene fluorophore which exhibits selective turn on response for mercury in NaCl containing aqueous solution. The chemosensor aggregates mercury ions which resulted in the emission at the wavelength of 470 nm [95]. This fluorescent sensor has the detection limit of 5.3 nM for mercuric ions which was remarkably lower than the permissible limit of mercury in drinking water. Development of these biosensors for heavy metal detection is reliable and durable which encourage the pragmatic aspects of multisensory approaches.

## 6. Scope of bioengineered char as a sustainable mode for removal of heavy metals

In the context of eliminating heavy metals contaminants from soil, Biochar (BC) offers materialized solution to greater extents. Biochar is a porous black-carbon enriched material obtained from pyrolysis or incomplete combustion of organic waste materials such as agricultural waste, slaughter waste, and activated sludge in limited oxygen supply [25,96,97]. It has proven its robust candidature in heavy metal removal with promising features like high aromaticity, low manufacturing cost, eco-friendly nature, thermal and mechanical stability, along with abundant availability of raw material in nature [25,26].

The intrinsic characteristics of biochar such as (i) high surface area (ii) porous structures (iii) multiple functional groups availability (iv) large pore size availability (v) higher affinity (vi) reusability (vii) strong magnetic properties (viii) high permeability (ix) environment friendly nature makes it a versatile candidate for bioenergy production, carbon sequestration, soil fertility or quality enhancement-cum-remediation, nutrient retention, crop yield enhancement and environment indemnification along with the superior quality of heavy metal absorption from soil and aqueous waste phases [96,98,99]. Biochar adopts different mechanisms like electrostatic interaction, co-precipitation, physical adsorption, surface complexation, ion exchange, pie-pie electron acceptor interaction, or a combination of these for removal of heavy metals from soil or aqueous waste [26,100]. The functional properties of biochar were influenced by its raw material and synthesis conditions. Generally, on average biochar was reported to improve the soil aggregation by 16.4% irrespective of soil, field, and experimental conditions and parameters [101].

Magnetic biochar composite (MBC) synthesized from pine bark waste and CoFe_2_O_4_ using facile fabrication method exhibited excellent sorption performance as per Langmuir equation at pH 4–5 for removal of Cd(II) and Pb(II) ions at industrial scale [96]. In MgO-coated biochar obtained from watermelon rind (MWRB) at 600°C with a fixed Mg/feedstock ratio of 2.51%, a maximum BET surface area of 293 m^2^/g was observed. Pb removal capacity of MWRB increases with increasing content of Mg from 1.52% to 10.1%. At 10.1% Mg concentration, 558 mg/g Pb removal was recorded. It reflects 208% improvement in Pb remediation as compared to 181 mg/g Pb removal from unmodified watermelon rind biochar produced at similar conditions [102].

Immobilization of *Brassica* bagasse biochar on heavy metal contaminated soil significantly reduces Pb (17.3–49.1%), Cu (15–38%), and Cd (62–76%) content through direct adsorption using physical adsorption, precipitation, ion exchange, electrostatic attraction, and complexation; or by improving the physicochemical properties (CEC, pH, organic and mineral matter content) and adsorption capacity of the soil [25]. Biochar produced by pyrolysis of *Oiltea camellia* shells waste at 500°C in the presence of sodium silicate exhibited remarkably improved absorption capabilities for Cd from contaminated soil at pH >5 and from wastewater. Silicate-modified biochar improves specific surface area (~45–112%), porosity of biochar (~5–12%), and internal Cd diffusion on biochar. As per XPS and FTIR analysis the prominent mechanism involved in Cd sorption and heavy metal irradiation process were Na^+^ ion exchange, surface precipitation (CdSiO3 or Cd2SiO4, CdCO3), C = C π electrons coordination, and complexation of carboxyl and C-Si-O groups [103].

Biochar colloids-mycelial pellets (BC-MP) produced by biological assembly method had improved performance of biomass stability, efficacy in heavy metal removal (57.66%), and maximum Cd (II) adsorption capacity (2.04 mg/g) due to the synergistic effect of mycelial pellets and biochar colloids. BC-MP offers an extended surface area for heavy metal attachment, multi-sorption sites for electrostatic interaction, physical adsorption, and H-bond formation between biochar colloids and extracellular polymers [104]. The effects of different concentrations *viz*., 2.5%, 5%, and 10% of lychee biochar on accumulation and distribution of Pb, Cd, Zn, and As in the biomass of sunflower plants (*Helianthus annuus*) and their concentrations in the rhizosphere soil was studied extensively. It was found that with increasing concentration of biochar, concentration of Pb, Cd, and As in the receptacles and leaves of sunflower plants increased by 22.9–58.9%, 67.9–110%, and 15.8–42.3% respectively while in roots, stems, and seeds their concentration was significantly low in comparison to the control. Zn content in sunflower plant was decreased by 13.8–37.2% due to the antagonistic effect of Cd, As, and Pb on Zn. The treated sunflower plants were effective in reducing the concentration of As, Zn, Pb, and Cd to 4.35, 8.17%, 12.4, and 11.0 respectively from contaminated soil as compared to the concentrations of heavy metals before sunflower planting i.e. 40.6%, 31.6%, 35.4%, and 30.8%, respectively [105]. Li et al., (2020) modified *Enteromorpha prolifera* biochar with different chemical reagents such as H_3_PO_4_, ZnCl_2_, and KMnO_4_ to evaluate the Cd(II) removal efficacy of biochar after chemical treatment. It was found that H_3_PO_4_ modified biochar considerably increases the adsorption capacity 423 mg/g of Cd(II) from wastewater. Biochar modified with phosphoric acid was reported to be very fast in response as it reached at saturation point for Cd(II) adsorption within 1 h [106].

Biochar prepared by aerobic/anaerobic hybrid calcination of *Eichhornia crassipes* had shown excellent adsorption capacities for Pb^2+^ (0.57 mmol/g), Cd^2+^ (0.44 mmol/g), Cu^2+^ (0.41 mmol/g) and Zn^2+^ (0.48 mmol/g) at 30°C. It was found to reach an adsorption equilibrium within 30 min of treatment [107]. Biochar has effectively attracted the focus of research community due to its heavy metal scavenging properties and sorptive behavior. It was concluded from Langmuir adsorption model that biochar adsorption capacities for lead(II) were maximum 109.9–256.4 mg/g to that of 29.5–42.7 mg/g cadmium(II), 18.5–39.4 mg/g copper(II), and 40.2–64.1 mg/g nickel(II). Biochar produced from rice straw and pulp mill sludge in the vicinity of carbon dioxide and nitrogen as the purging gas showed highest lead(II) adsorption capacities of 256.4 and 133.3 mg/g, respectively in N gas containing biochar while 250.0 and 109.9 mg/g, respectively for biochar produced with CO2 gas. This process yielded 30–62% adsorption of heavy metal in 1 h of treatment (Islam et al., 2021). It was found that for assessing soil aggregation wet sieving method (18.2%) was superior to dry sieving method (4.05%) of biochar. Neutral to acidic soil biochar produce intensified aggregation while in alkaline soil it is not much effective to produce any remarkable change. In loam textured soil biochar amendment produced 19.9% aggregation compared to 13.4% in sandy soils [101].

## 7. Knowledge gaps and Perspectives

Technical and financial complexities have made the mitigation of heavy metals a challenging task. The traditional approaches used for the heavy metals removal have become outdated because of several drawbacks (Table 3). The methods such as membrane filtration, Ion exchange, adsorption, Coagulation and flocculation, and even the eco-friendly biological methods have few disadvantages as shown in Table 2 [33,108–110]. The majority of these processes have become outdated with low public acceptability owing to their high cost, incomplete removal of heavy metal, generation of secondary pollution, and requirement of additional treatment. Among these, biological remediation is most preferred. The biological remediation including plants and/or microorganisms has certain limitation but are most acceptable owing to their eco-friendly advantages [111,112]. In addition to this the introduction of biochar was costly, and to overcome these problems they have been implemented with magnetic metal oxides which allow its reusability and thus will affect the acceptability.

**Table 2.** Microalgae used for remediation of different heavy metals

**Microalgae name**	**Heavy metal**	**Initial concentration (mg/L)**	**Final concentration** **(mg/L)**	**Reference**
Chlorella vulgaris	Cd	25–150	58.4	[^115]^
Chlorella vulgaris	Fe	27.14	0.47	[^82]^
Chlorella vulgaris	Zn	3.90	0.02	[^82]^
Chlorella vulgaris	Cu	4.03	0.12	[^82]^
Cladophora fascicularis	Cu	12.7–254.2	70.54	[^116]^
Desmodesmus pleiomorphus	Cd	0.5–5	61.2	[^117]^
Chlorella vulgaris	Cd	0.05	0.00	[^82]^
Ecklonia maxima	Cd, Cu	–	–	[^118]^
Chlorella vulgaris	As	0.08	0.00	[^82]^
Chlorella minutissima	Zn, Mn, Cd, Cu	–	–	[^119]^
Chondrus crispus	Cd, Zn	10–150	75.2, 45.7	[^120]^

**Table 3.** Limitation and acceptability of different remediation approaches

Method	Drawbacks	Acceptability	Reference
Adsorption	Requirement of host material, Generation of secondary pollution.	Very low	[^110]^
Coagulation and flocculation	Sludge generation, Requirement of additional treatment.	Low	[^109]^
Membrane filtration	High operation pressure, Low permeate flux.	Very low	[^33]^
Ion exchange	Low selectivity, Less economic, Generation of secondary pollution.	Very low	[^108]^
Immobilization of metals	Does not provide permanent solution, Require marked demand to dispose the immobilized materials.	High public acceptability	[^112]^
Phytoremediation	Low biomass, requirement for plant growth promoting rhizospheric bacteria; time-consuming process.	Low-medium public acceptability	[^121]^
Microbial assisted phytoextraction	Dependent on soil, plant, metal, and microorganism type.	Very high public acceptability	[^112]^
Microbial remediation	Dependent on microorganism employed, need optimum environmental conditions, high concentration of toxic metals cal kills microbes, require nutrient addition	High public acceptability	[^47]^

To overcome the technical and practical limitations with the use of bioremediation technique, several steps can be implemented. This includes the systematic management and uses on urban or industrial sites with low contamination in order to decrease the metal content while simultaneously increasing the fertility of the soil. Furthermore, employing transcriptomic approach can enhance the bioremediation effectiveness and site implementation. The introduction of genes pertaining to the resistance/tolerance, and uptake of these contaminants can further increase the bioremediation potential by plants and microorganisms. An ideal plant with characteristics branched root system, good phytoremediation capacity, tolerance of harsh environmental conditions, easy to harvest, increased potential to absorb, mobilize, sequester, and transfer metals can be generated employing genetic engineering approach [113] and introducing such plant at contaminated sites may significantly enhance the bioremediation process. A similar approach can be followed for the developments of engineered microorganisms with enhance synthesis of specific enzymes needed for adhesion, transformation, and mineralization of inorganic pollutants. To generate such results the knowledge of the genome is crucial, which can be attained using omics approaches [114]. Genomics, transcriptomic, proteomics, and metabolomics together will unfold the genetic map toward the improvement of species for its employment in the remediation of heavy metals.

## 8. Conclusions

Heavy metals are among the most toxic and deleterious pollutants. Biological or anthropogenic sources are predominant reasons for their environment occurrence, which adversely affect the aquatic and terrestrial ecosystem because of their properties such as hazardous in nature, bioaccumulation, and persistence. Over the period, microorganisms and plants have evolved with mechanisms to reduce the level of these pollutants. The use of microbial enzymes has shown significant potential in combating heavy metal pollution. The development of sensors and advance detection methods has made it possible to monitor and quantify the level of heavy metals in biotic and abiotic environments with better efficiency and reliability. The recent advancements with the use of waste derived biochar for the remediation of heavy metal polluted environments have opened new avenues toward sustainable approach in heavy metal removal. The state-of the art information about technological advancements provided in this article would be helpful to the researchers and academicians working in heavy metal detection and remediation.
